# Impact of age on the diagnostic performance of unstimulated salivary flow rates and salivary gland ultrasound for primary Sjögren's syndrome

**DOI:** 10.3389/fmed.2022.968697

**Published:** 2022-10-20

**Authors:** Kyung-Ann Lee, Se-Hee Kim, Hae-Rim Kim, Hyun-Sook Kim

**Affiliations:** ^1^Division of Rheumatology, Department of Internal Medicine, Soonchunhyang University Seoul Hospital, Soonchunhyang University School of Medicine, Seoul, South Korea; ^2^Division of Rheumatology, Department of Internal Medicine, Konkuk University Medical Center, Konkuk University School of Medicine, Seoul, South Korea

**Keywords:** primary Sjögren's syndrome, salivary flow rate, diagnosis, classification, ultrasound, age

## Abstract

**Background:**

Age-related changes and different patterns of salivary gland abnormalities according to age may affect the diagnostic performance of unstimulated salivary flow rate (USFR) and salivary gland ultrasound (SGUS) for primary Sjögren's syndrome (pSS). We aimed to evaluate the threshold and diagnostic performance of USFR and whether incorporating SGUS or replacing USFR with SGUS affects the performance of the ACR/EULAR criteria for pSS according to age.

**Materials and methods:**

This medical chart review study included patients with suspected pSS who completed evaluations for pSS. Patients were classified based on age at pSS evaluation: elderly (≥65 years), middle-aged (40–64), and young (< 40). The USFR's optimal thresholds were evaluated using the ROC curve. The diagnostic performances of the USFR and modified ACR/EULAR criteria were compared.

**Results:**

In total, 239 pSS patients and 92 patients with idiopathic sicca syndrome were included. The cut-off of USFR ≤ 0.1 mL/min was irrelevant to age, demonstrating the best sensitivity (44.3–53.0%) and specificity (74.1–90.9%). SGUS had a significantly better AUC than USFR in the young (*p* < 0.01) and middle-aged groups (*p* < 0.01). The middle-aged group demonstrated better diagnostic performance of the ACR/EULAR criteria incorporating SGUS (AUC 0.957) (*p* < 0.01) and criteria replacing USFR with SGUS (AUC 0.957) (*p* < 0.001) compared to the original criteria (AUC 0.916). In the young and elderly groups, adding SGUS to the ACR/EULAR criteria or replacing USFR with SGUS did not significantly increase the AUC.

**Conclusions:**

The thresholds of USFR ≤ 0.1 mL/min was optimal, irrespective of age. Using SGUS can improve diagnostic accuracy of ACR/EULAR criteria by supplementing the USFR, especially in middle-aged patients.

## Introduction

Primary Sjögren's syndrome (pSS) is a systemic autoimmune disease characterized by exocrinopathy, resulting in dryness of the mouth and eyes (1, 2). The 2016 American College of Rheumatology (ACR)/European League against Rheumatism (EULAR) criteria use whole unstimulated salivary flow rate (USFR) and minor salivary gland biopsy as measures of the salivary gland involvement (3). Scintigraphy and sialography data were omitted from the 2016 ACR/EULAR classification criteria. To overcome the limitations of the absent salivary gland imaging technique in these classification criteria, incorporating salivary gland ultrasound (SGUS) in the ACR/EULAR criteria has been reported to improve diagnostic performance (4–6).

The USFR test is an easy method for measuring salivary glandular function (7). Although standardized methods for USFR have been described specifically (8), a threshold of 0.1 ml/min has not been validated in the 2016 ACE/EULAR (3) and 2002 American-European Consensus Group classification criteria (9). A recent cohort study reported that a threshold of 0.2 mL/min increased the sensitivity and specificity in women aged < 50 years and in men. In women aged ≥50 years, the USFR had poor diagnostic performance, suggesting that the performance of the USFR in diagnosing pSS varies according to age and sex (10). However, in a previous study, the numbers of young and elderly patients were limited. Histological studies have demonstrated age-induced changes in the salivary gland structure, including an increase in fibrotic tissue, duct dilatation, and a decrease in acinar volumes (11). A decrease in salivary gland function in healthy individuals has also been reported as the age increased (12). Therefore, it is necessary to verify whether the cut-off of the USFR needs to be changed according to the age group by conducting a study including more young and elderly patients with suspected pSS.

All studies incorporating SGUS in the existing classification criteria have reported an improvement in diagnostic accuracy with increased sensitivity and slightly decreased specificity, regardless of the pSS classification (13). Distinct clinical and serological features of early- and elderly-onset pSS have been previously reported. Although the subjective oral dryness and atrophic changes on SGUS were more frequent in elderly pSS patients, young pSS patients had higher SGUS scores and more frequent enlargement of salivary glands (14–16). Different patterns of salivary gland abnormalities according to age may affect the diagnostic performance of the classification criteria for pSS when incorporating SGUS.

The objectives of our study were (1) to evaluate the threshold and diagnostic performance of USFR and (2) to assess whether the addition of SGUS or replacement of USFR by SGUS affects the performance of the ACR/EULAR classification criteria for pSS in patients with suspected pSS according to age.

## Materials and methods

### Study design and population

This was a medical chart review study of patients who presented with sicca symptoms or diverse extra-glandular symptoms leading to a suspected pSS conducted two centers in South Korea between September 2016 and August 2021. Patients with pSS, and idiopathic Sicca syndrome as controls were enrolled. The definitive diagnosis of pSS was made in accordance with the 2016 ACR/EULAR classification criteria. Patients who completed at least the anti-SSA/Ro, USFR, Schirmer tests, and SGUS were included. Patients who were diagnosed with idiopathic Sicca syndrome, which is characterized by non-immune mediated manifestations of dry eyes and mouth, served as controls.

The ocular staining score (OSS) and focus score (FS) were missing in ~25 and 20% of our population, respectively, because OSS was performed at only one institution, and FS was performed according to the judgment of rheumatologists. OSS is a minor variable (1 point) in the 2016 ACR/EULAR classification criteria; therefore, even when OSS was missing, the pSS diagnosis was considered for a total score ≥4 points according to the 2016 ACR/EULAR classification criteria. Patients with anti-SSA/Ro and other minor variables (USFR, Schirmer tests, and/or OSS) were considered to have pSS, even when FS was missing. Patients with secondary SS or those who met the exclusion criteria according to the 2016 ACR/EULAR classification criteria 3, uncertain diagnosis, incomplete medical records, or evaluations for pSS were excluded. For example, missing OSS or FS data could change the pSS diagnosis in patients with a score of 3 according to the ACR/EULAR classification. In such cases, we excluded them from our study population due to uncertainty of diagnosis. In controls with positive anti-SSA/Ro and/or anti-SSB/La, only patients who completed the evaluation for pSS including minor salivary gland biopsy and OSS were included in this study.

### Clinical, laboratory, and histologic evaluation

Patients were divided into three groups according to age at the time of diagnosis: elderly (≥65 years), middle-aged (40–64 years), and young (< 40 years) (15). The following data were collected during diagnosis: age, sex, duration of sicca symptoms, history of dry eyes/mouth, autoantibodies (antinuclear antibody, anti-SSA/Ro, and anti-SSB/La), Schirmer's test result (≤ 5 mm/5 min on at least one side is abnormal), and whole USFR, OSS, FS (17), and presence of comorbidities (including diabetes, hypertension). The USFR was assessed by questioning the patient to expectorate all saliva over a period of 15 min without gustatory provocation, as recommended (8).

Additionally, we investigated the disease-related extra-glandular involvement of pSS at the time of diagnosis as follows: lymphadenopathy, arthritis, peripheral nervous system involvement, central nervous system involvement, renal involvement), interstitial lung disease (ILD) confirmed by high-resolution computed tomography, and purpura (18). To investigate the biological activity in patient with pSS, complement levels (C3, C4), rheumatoid factor, and immunoglobulin G was also assessed at the time of diagnosis.

### SGUS evaluation

Two rheumatologists (KA Lee, 6 years of experience in SGUS, and SH Kim, 3 years of experience in SGUS) independently reviewed the static images of SGUS at the time of diagnosis, blinded to the patients' final diagnoses. Each of the four salivary glands was individually graded according to the 2019 Outcome Measures in Rheumatology (OMERACT) US scoring system (19). The grades were as follows: grade 0, normal parenchyma; grade 1, minimal change, mild inhomogeneity without anechoic/hypoechoic areas or fatty change defined as homogeneous hyperechoic glands compared with the adjacent tissue; grade 2, moderate change, moderate inhomogeneity with focal anechoic/hypoechoic areas; grade 3, severe change, diffuse inhomogeneity with anechoic/hypoechoic areas occupying the entire gland surface or fibrous echostructures. In case of disagreement between the rheumatologists, the results obtained by the more experienced rheumatologist were used to analyse the diagnostic performance. A score ≥ 2 for the most affected salivary gland defined SGUS as compatible with a pSS diagnosis (4). A total of 0–12 total SGUS scores were calculated as the sum of the four glands. Furthermore, each pair of glands was scored separately from 0 to 6 (20).

### Statistical analysis

All statistical analyses were performed using Rex (version 3.6.0, RexSoft Inc., Seoul, Korea). Data were expressed as the median (Q1, Q3) or mean (standard deviation, SD) for continuous variables, as appropriate, and as absolute frequencies and percentages (%) for qualitative variables. Data were compared using the unpaired Student's *t*-test, Mann–Whitney U test, chi-square test, and Fisher's exact test, as appropriate. Spearman's rank correlation was used to assess the USFR correlation with age, SGUS scores, and FS. Inter-observer agreement were estimated by the kappa (k) coefficient for OMERACT SGUS score [normal-appearing scores (0–1) vs. pathological scores (2–3)]. Kappa values of 0–0.20 were considered slight, 0.21–0.40 fair, 0.41–0.60 moderate, 0.61–0.80 substantial, and 0.81–1.00 almost perfect (21). Using the clinical diagnosis as the gold standard, we examined the diagnostic performance of USFR and the original and modified ACR/EULAR criteria with the inclusion of SGUS or replacement of USFR with SGUS. Using the receiver operating characteristic (ROC) curve, the USFR thresholds were evaluated for the entire population and patients according to age groups. On the ROC curve, the optimal cut-off value producing the best combination of sensitivity and specificity was located near the upper-left corner of the curve. We estimated the sensitivity, specificity, positive predictive value (PPV), and negative predictive value (NPV) for each diagnostic method and criteria set according to the age group. The discriminant power of each criterion set was compared using the area under the ROC curve (AUC) analyses, and Bonferroni corrections for multiple comparisons were performed. The results were considered statistically significant if *p* < 0.05.

## Results

### Baseline characteristics of the study population

A total of 239 pSS patients and 92 controls were enrolled with a mean (SD) age of 55.4 (14.7) and 58.2 (13.2) years, respectively. The baseline characteristics of the three groups are summarized in Table 1. The mean (SD) USFR was 0.21 (0.2) ml/min in the pSS groups and 0.34 (0.29) ml/min in the control group (*p* < 0.001). As expected, pSS had significantly higher positivity for USFR, OSS, autoantibodies, FS, and SGUS scores than the controls. There was no significant difference between the pSS and control groups concerning comorbidities. In the pSS group, the positivity of the USFR and mean USFR was not significantly different among the three age groups. Young pSS patients had a significantly higher prevalence of anti-SSA/Ro (*p* < 0.001) and lower positivity in the Schirmer's test (*p* < 0.001) than the other pSS groups.

**Table 1:** Clinical and laboratory characteristics of the study population.

	**pSS**				**Control**				***P-*value**
	**Total** **(*n* = 239)**	** < 40 years** **(*n* = 40)**	**≥40 and < 65 years** **(*n =* 133)**	**≥65 years** **(*n =* 66)**	**Total ** **(*n =* 92)**	** < 40 years** **(*n =* 11)**	**≥40 and < 65 years** **(*n =* 50)**	**≥65 years** **(*n =* 31)**	**Total pSS vs. controls**
Age, years	55.4 (14.7)	30.3 (5.7)	54.8 (6.8)	71.7 (4.5)	58.2 (13.8)	31.4 (5.2)	55.3 (5.9)	72.3 (5.7)	0.114
Female, *n* (%)	223 (93.3)	35 (87.5)	125 (93.9)	63 (95.4)	77 (83.7)	11 (100)	43 (86)	23 (74.1)	**0.003**
Xerostomia, *n* (%)	209 (87.8)	29 (72.5)	118 (88.7)	62 (93.9)	81 (88.0)	8 (72.7)	46 (92.0)	27 (87.1)	0.821
Xerophthalmia, *n* (%)	213 (89.8)	31 (77.5)	122 (91.7)	60 (90.9)	81 (88.0)	11 (100)	43 (86.0)	27 (87.1)	0.692
Duration of sicca symptoms, years	5.1 (5.2)	2.5 (1.8)	5.4 (5.3)	5.6 (5.6)	3.7 (4.3)	2.71 (3.4)	3.5 (3.6)	4.1 (4.9)	**0.030**
Abnormal Schirmer's test, *n* (%)	193 (80.7)	20 (50)	115 (86.4)	60 (90.9)	61 (66.3)	1 (9.09)	38 (76)	21 (67.7)	**0.001**
USFR, ml/min	0.21 (0.2)	0.23 (0.23)	0.21 (0.21)	0.19 (0.18)	0.34 (0.29)	0.32 (0.19)	0.36 (0.34)	0.29 (0.24)	**0.0002**
USFR ≤ 0.1 ml/min, *n* (%)	112 (46.8)	18 (45.0)	59 (44.3)	35 (53.0)	20 (21.7)	1 (9.09)	11 (22)	8 (25.8)	**< 0.001**
OSS ≥ 5, *n* (%)	45/119 (37.8)	2/11 (18.1)	23/69 (33.3)	20/39 (51.2)	1/48 (2.1)	0/5 (0)	1/24 (4.17)	0/19 (0)	**< 0.001**
Positive anti-Ro/SSA, *n* (%)	176 (73.6)	35 (87.5)	104 (78.2)	37 (56.0)	6 (6.5)	3 (27.2)	3 (6)	0 (0)	**< 0.001**
Positive anti-La/SSB, *n* (%)	79/238 (33.2)	21/39 (53.8)	48 (36.1)	10 (15.1)	4 (4.3)	2 (18.1)	2 (4)	0 (0)	**< 0.001**
FS ≥ 1, *n* (%)	110/123 (89.4)	6/8 (75.0)	64/70 (91.4)	40/45 (88.8)	2/71 (2.8)	0/9 (0)	0/38 (0)	2/24 (12.5)	**< 0.001**
Max SGUS OMERACT grade ≥ 2, *n* (%)	141 (59.0)	33 (82.5)	82 (61.7)	26 (39.4)	14 (15.2)	1 (9.1)	7 (14)	6 (19.3)	**< 0.001**
Total SGUS OMERACT grades, median (Q1,Q3)	7 (2, 10)	8 (6, 9)	7 (0, 10)	6.0 (2, 10)	2 (0, 3)	0 (0,2)	2 (0, 3)	2 (0, 2.75)	**< 0.0001**

Values are expressed as mean (standard deviation) unless otherwise stated. pSS, primary Sjogren's syndrome; Ns, non-significant; USFR, unstimulated salivary flow rate; ANA, antinuclear antibody; RF, rheumatoid factor; SGUS, salivary gland ultrasound. Bold values denote statistical significance.

Elderly patients with pSS presented a significantly higher frequency of xerostomia (93.9 vs. 88.7 vs. 72.5%, *p* = 0.042). xerophthalmia (90.9 vs. 91.7 vs. 77.5%, *p* = 0.007), ILD (19.2 vs. 15.0 vs. 2.5%, *p* = 0.045), and lower frequency of lymphadenopathy (10.6 vs. 13.5 vs. 20%, *p* = 0.002) than middle-aged and young patients. On the other hand, we did not find any statistical difference between the three groups regarding arthritis, involvement of PNS, and CNS, and purpura. In terms of biological parameters, prevalence of low C4 levels was higher in young patients 24.2%) than middle-aged (7.6%) and elderly patients (3.7%) (*p* = 0.004).

Although the controls had a negative correlation between the USFR and age (*r* = −0.229, *p* < 0.05), these correlations were not observed in the pSS group. A significant negative correlation between the USFR and SGUS scores (*r* = −0.479, *p* < 0.001) was found in the pSS group.

### Threshold and diagnostic performance of USFR according to age group

**Figure 1:**
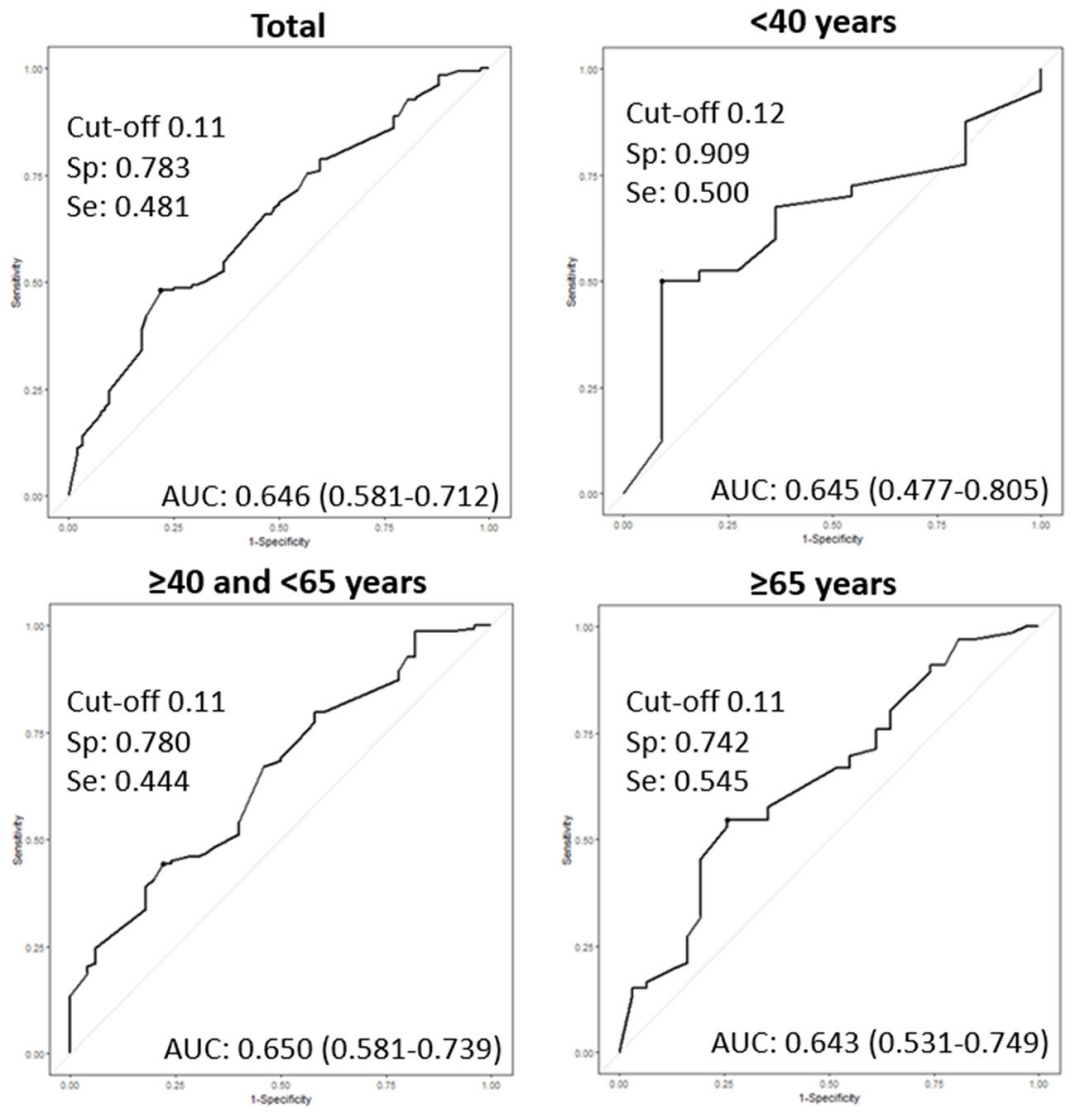
Receiver operating characteristic curves (ROC) of the unstimulated salivary flow rate (USFR) for diagnosis of primary Sjogren's syndrome (pSS) according to age groups. AUC: area under the curve, Sp: specificity, Se: sensitivity.

Using the ROC curve for USFR, the optimal cut-off producing the best sensitivity and specificity was observed as 0.11 ml/min in the entire population. The best threshold was 0.11 ml/min for middle-aged and elderly groups, and 0.12 ml/min for the young group (Figure 1).

Table 2 shows the diagnostic performance of the USFR at the threshold of 0.1 and 0.2 mL/min. By setting a USFR cut-off value of 0.1 ml/min [AUC 0.62 (95% CI: 0.57–0.68)] (*p* < 0.001), USFR had 47.7% (95% CI: 41.2–54.2) sensitivity, and 78.2% (65.7–86.1) specificity in the entire population. Among the three age groups, young (≤ 40 years) patients had the highest AUC [0.70 (0.58–0.82)] than middle-aged [0.61 (0.54–0.68)] (*p* < 0.05) and elderly patients [0.63 (0.53–0.73)] (*p* < 0.05). A threshold of 0.2 mL/min demonstrated an increase in sensitivity of 15.0, 10.0, 19.6, and 9.1% in the entire population, young patients, middle-aged patients, and elderly patients, respectively. However, the specificity, PPV, NPV, and AUC decreased in the entire population and in the three age groups. Furthermore, the AUC of the USFR at a cut-off of 0.2 mL/min was only significant in middle-aged patients [AUC 0.58 (95% CI: 0.50–0.67)] (*p* < 0.05). The AUC of the young and elderly groups was 0.61 (95% CI: 0.45–0.78) (*p* = 0.084) and 0.55 (95% CI: 0.44–0.65) (*p* = 0.234), respectively.

**Table 2:** Performance of the unstimulated salivary flow rate tests for diagnosing primary Sjögren's syndrome according to age group.

**Cut-off (mL/min)**	**Sensitivity**	**Specificity**	**PPV**	**NPV**	**AUC**
**Whole population**
≤ 0.1	47.7 (41.2–54.2)	78.2 (65.7–86.1)	85.0 (77.8–90.6)	36.5 (29.8–43.6)	0.62 (0.57–0.68)
≤ 0.2	62.7 (56.3–68.9)	53.2 (42.5–63.7)	77.7 (71.1–83.3)	35.5 (27.5–44.1)	0.58 (0.52–0.63)
**Young** **(< 40 years)**
≤ 0.1	50.0 (33.8–66.2)	90.9 (58.7–99.7)	95.2 (76.1–99.8)	33.3 (17.2–52.8)	0.70 (0.58–0.82)
≤ 0.2	60.0 (43.3–75.1)	63.6 (30.7–89.0)	85.7 (67.3–95.9)	30.4 (13.2–52.9)	0.61 (0.45–0.78)
**Middle-aged** **(40–64 years)**
≤ 0.1	44.3 (35.7–53.2)	78.0 (64.0–88.4)	84.2 (73.6–91.8)	34.5 (25.8–44.0)	0.61 (0.54–0.68)
≤ 0.2	63.9 (55.1–72.0)	54.0 (39.3–68.1)	78.7 (69.7–86)	36 (25.2–47.9)	0.58 (0.50–0.67)
**Elderly** **(≥65 years)**
≤ 0.1	53.0 (40.3–65.4)	74.1 (55.3–88.1)	81.4 (66.6–91.6)	42.5 (29.2–56.7)	0.63 (0.53–0.73)
≤ 0.2	62.1 (49.3–73.7)	48.3 (30.1–66.9)	71.9 (58.4–83.0)	37.5 (22.7–54.2)	0.55 (0.44–0.65)

Values are expressed as % (95% confidence interval). PPV, positive predictive value; NPV, negative predictive value; AUC, area under the receiver operating characteristic curve; CI, confidence interval.

### Comparison of the performance of USFR with other diagnostic methods according to age group

Table 3 presents the diagnostic performance of the current items included in the 2016 EULAR/ACR criteria and SGUS OMERACT scores according to age group. Except for the anti-SSA/Ro and FS, SGUS demonstrated the best AUC in the entire population and in all three age groups. In the ROC comparison analysis, SGUS OMERACT scores had significantly better AUC in the entire population [0.772 (0.729–0.822)] (*p* < 0.001), the young group [0.867 (0.759–0.974)] (*p* < 0.01), and the middle-aged group [0.736 (0.668–0.804)] (*p* < 0.01) than the USFR. In the elderly group, SGUS had a higher AUC [0.703 (0.606–0.801)] than USFR; however, the difference was not significant (*p* = 0.266). Inter-observer agreement was substantial for OMERACT SGUS score (kappa value: 0.779; 95% CI 0.672–0.886).

**Table 3:** Diagnostic performance of items in the ACR/EULAR criteria of primary Sjögren's syndrome and salivary gland ultrasound according to the age group.

	**Sensitivity**	**Specificity**	**AUC**
**Anti-SSA/Ro**
Whole	73.6 (67.5–79.1)	93.4 (86.3–97.5)	0.835 (0.797–0.873)
< 40	87.5 (73.2–95.8)	72.7 (39.0–93.9)	0.801 (0.653–0.948)
≥40 and < 65	78.2 (70.2–84.8)	94 (83.4–98.7)	0.861 (0.812–0.909)
≥65	56.0 (43.3–68.2)	100 (88.7–100)	0.780 (0.72–0.840)
**Max OMERACT US grade** **≥2**
Whole	64.2 (57.6–70.3)	84.2 (74.0–91.5)	0.742 (0.69–0.793)
< 40	82.5 (67.2–92.6)	90.9 (58.7–99.7)	0.867 (0.759–0.974)
≥40 and < 65	61.5 (52.6–69.9)	85.7 (71.4–94.5)	0.736 (0.668–0.804)
≥65	60.0 (47.1–71.9)	80.7 (60.6–93.4)	0.703 (0.606–0.801)
**Focus score** **≥1/4 mm**^**2**^
Whole	89.4 (82.6–94.2)	94.7 (73.9–99.8)	0.92 (0.862–0.979)
< 40	75.0 (34.9–96.8)	100 (29.2–100)	0.875 (0.714–1.000)
≥40 and < 65	91.4 (82.2–96.7)	100 (71.5–100)	0.957 (0.924–0.99)
≥65	88.8 (75.9–96.2)	87.5 (47.3–99.6)	0.881 (0.75–1.00)
**Schirmer** **≤5 mm/5 min**
Whole	80.7 (75.1–85.5)	33.7 (24.1–44.3)	0.572 (0.517–0.626)
< 40	45.0 (29.2–61.5)	81.8 (48.2–97.7)	0.634 (0.491–0.776)
≥40 and < 65	86.4 (79.4–91.7)	24.0 (13.0–38.1)	0.552 (48.5–61.8)
≥65	90.9 (81.2–96.5)	32.2 (16.6–51.3)	0.615 (0.525–0.706)
**Ocular staining score**
Whole	37.8 (29.0–47.1)	97.9 (88.9–99.9)	0.678 (0.63–0.726)
< 40	18.1 (2.2–51.7)	100 (47.8–100)	0.59 (0.471–0.71)
≥40 and < 65	33.3 (22.4–45.7)	95.8 (78.8–99.8)	0.645 (0.576–0.715)
≥65	51.2 (34.7–67.5)	100 (82.3–100)	0.756 (0.676–0.835)

Values are expressed as % (95% confidence interval). PPV, positive predictive value; NPV, negative predictive value; AUC, area under the receiver operating characteristic curve.

### Diagnostic performance of ACR/EULAR criteria with the addition of SGUS and with replacement of USFR with SGUS according to age group

In the entire population and middle-aged group, adding SGUS to the ACR/EULAR criteria with a weight of 1 point as proposed by van Nimwegen et al. (22) significantly increased the diagnostic performance. In the entire population, ACR/EULAR with SGUS demonstrated a better AUC [0.937 (95% CI: 0.937)] (*p* < 0.01) and increased sensitivity [92.0% (88.0–95.1)] than the original ACR/EULAR classification criteria [AUC: 0.909 (0.883–0.935)] [sensitivity: 85.3% (80.2–89.5)] (Table 4). The ACR/EULAR criteria with SGUS replacing USFR also increased the AUC [0.931 (0.906–0.962)] and sensitivity [90.7% (86.2–94.0)]; however, the difference was not statistically significant.

**Table 4:** Performance of the original and modified ACR/EULAR criteria incorporating SGUS and replacing USFR by SGUS according to the age group.

	**Sensitivity**	**Specificity**	**AUC**	*P*-value*
**Whole population**
Original ACR/EULAR	85.3 (80.2–89.5)	96.7 (90.7–99.3)	0.909 (0.883–0.935)	
ACR/EULAR + SGUS	92.0 (88.0–95.1)	95.6 (89.1–98.7)	0.937 (0.908–0.96)	**0.006**
SGUS replacing USFR	90.7 (86.2–94.0)	95.6 (89.1–98.7)	0.931 (0.906–0.962)	0.163
**Young (< 40 years)**
Original ACR/EULAR	75.0 (58.8–87.3)	100 (71.5–100)	0.868 (0.789–0.934)	
ACR/EULAR + SGUS	85.0 (70.1–94.2)	90.0 (55.5–99.7)	0.871 (0.764–0.973)	1.000
SGUS replacing USFR	78.9 (62.6–90.4)	90.0 (55.5–99.7)	0.844 (0.711–0.934)	1.000
**Middle-aged (40–64 years)**
Original ACR/EULAR	87.2 (80.3–92.3)	96.0 (86.2–99.5)	0.916 (0.868–0.953)	
ACR/EULAR + SGUS	95.4 (90.4–98.3)	96.0 (86.2–99.5)	0.957 (0.924–0.99)	**0.003**
SGUS replacing USFR	95.5 (90.4–98.3)	96.0 (86.2–99.5)	0.957 (0.918–0.980)	**< 0.001**
**Elderly (≥65 years)**
Original ACR/EULAR	87.8 (77.5–94.6)	96.7 (83.3–99.9)	0.923 (0.856–0.962)	
ACR/EULAR + SGUS	89.3 (79.3–95.6)	96.7 (83.3–99.9)	0.930 (0.888–0.969)	0.895
SGUS replacing USFR	87.8 (77.5–94.6)	96.7 (83.3–99.9)	0.923 (0.872–0.962)	1.000

Values are expressed as % (95% confidence interval). AUC, area under the receiver operating characteristic curve. ^*^P-value corrected by Bonferroni adjustment are presented. PPV, positive predictive value; NPV, negative predictive value. Bold values denote statistical significance.

Especially in the middle-aged group, ROC comparison analysis demonstrated a better diagnostic performance of both ACR/EULAR criteria with SGUS [AUC 0.957 (0.924–0.99)] (*p* < 0.01) and ACR/EULAR criteria with SGUS replacing USFR [0.957 (0.918–0.980)] (*p* < 0.001) than the original ACR/EULAR criteria [0.916 (0.868–0.953)]. In both young and old age groups, the modified ACR/EULAR criteria adding SGUS or replacing USFR with SGUS did not significantly increase the AUC.

## Discussion

This study investigated the optimal cut-off and diagnostic performance of the USFR for the diagnosis of pSS according to three age groups. The cut-off of USFR ≤ 0.1 mL/min was irrelevant to age, demonstrating the best discriminative ability. As a single diagnostic method, SGUS OMERACT scores had significantly better capacity than the USFR in the young and middle-aged groups. The 2016 ACR/EULAR incorporating SGUS and replacing USFR with SGUS revealed significantly better diagnostic utility for pSS in the middle-aged group but demonstrated comparable performance in the young and elderly groups.

The USFR is a non-invasive and easy method to diagnose pSS in clinical practice. In the 2016 ACR/EULAR classification criteria for pSS, the USFR was the sole item evaluating salivary glandular dysfunction (3). However, the USFR threshold of 0.1 mL/min was chosen based on a small study that included 25 patients with pSS (23). Histopathological and SGUS studies reported age related-changes in the salivary glands, including fibrosis, atrophy, and duct dilatations (11, 15). Conversely, young pSS patients had higher SGUS scores and more frequent enlargement of salivary glands, reflecting a severe phenotype of salivary glandular involvement. A recent study reported a threshold of the USFR to 0.2 mL in women < 50 years and men, and USFR had poor diagnostic performance in women ≥50 years. However, only 16 patients with pSS were included in the < 50 years group, and there were few elderly patients (10). Therefore, we assessed the cut-off of the USFR according to the three age groups. In particular, our study included more patients in their 20–30s and over 65 years of age.

In contrast to the controls, the pSS group did not demonstrate a negative correlation between age and USFR in this study. The pSS group revealed a negative correlation between the USFR and total SGUS scores, suggesting that salivary gland dysfunction was more influenced by inflammation and/or damage than the aging process. The young pSS group had the highest SGUS scores among the three groups, and these severe glandular changes could affect the similar mean USFR compared to the elderly group. The young pSS group had the largest AUC of USFR ≤ 0.1 mL/min with a high specificity of 90.9%. The low prevalence of positive USFR in the young controls (9%) could have affected these results. Contrary to the study by Lacombe et al. (10), a USFR threshold of 0.1 mL/min had a better diagnostic performance than 0.2 mL/min in all age groups. USFR threshold of 0.2 mL/min increased sensitivity by 9.1–19.6% but decreased specificity by 24–27.3% compared to 0.1 mL/min. In the elderly control group, more than half of the patients (16/31, 51.6%) had a USFR ≤ 0.2 mL/min. Our results confirmed the current threshold of USFR in the 2016 ACR/EULAR classification criteria for pSS (3).

Owing to the lack of imaging methods to visualize the major salivary glands, many researchers are interested in incorporating SGUS into the current ACR/EULAR criteria (4, 22, 24). A recent study demonstrated that the combination of positive SGUS and the presence of anti-SSA/Ro antibodies highly predicted the pSS classification according to the ACR/EULAR classification criteria (25). Based on this study, SGUS has been suggested as the first step in diagnosing pSS (13). For the first time, our study investigated the diagnostic performance of the modified ACR/EULAR classification criteria that added SGUS or replaced USFR with SGUS according to age groups. In this study, higher SGUS abnormalities, defined as a maximal OMERACT grade ≥2, were detected in young pSS patients (82.5%) than in middle-aged (61.5%) and elderly pSS patients (40%). Similar to previous studies (5, 6), the ACR/EULAR criteria with SGUS improved sensitivity in the young and middle-aged groups. However, owing to the low positivity of SGUS in elderly pSS patients, the ACR/EULAR criteria integrated with SGUS did not improve the performance. In elderly patients, considering the low prevalence of anti-SSA/Ro and positive SGUS findings in pSS and the relatively high positivity of USFR and Schirmer tests in controls, salivary gland biopsy remains an irreplaceable method for diagnosing pSS.

This study reported a much higher specificity of SGUS than USFR to diagnose pSS in young and middle-aged patients. In the middle-aged group, the modified ACR/EULAR classification criteria replacing USFR with SGUS revealed significantly higher AUC values than the original criteria, and also demonstrated comparable diagnostic performance in the young and elderly groups. van Nimwegen et al. reported that when SGUS replaced the USFR, ocular staining score, or Schirmer's test, performance remained, while the sensitivity of the criteria significantly decreased when SGUS replaced salivary gland biopsy or anti-SSA/Ro antibodies (22). Our results support the use of SGUS as a frontline method for the diagnosis of pSS, especially in young and middle-aged patients.

USFR measurement provides information on the salivary gland function, and EULAR recommends USFR as a determinant for deciding therapeutic interventions in pSS patients (26). Although various factors such as drugs, environmental temperature, diet, stimulation, circadian rhythm, and infections can affect the salivary flow rate (27, 28), SGUS is an imaging method to detect anatomical and morphological structures of salivary glands and has the advantage of not being affected by other conditions. Our study results provide evidence that incorporating SGUS in the ACR/EULAR criteria, rather than replacing USFR with SGUS, improves the diagnostic accuracy of the original criteria in all age groups. USFR and SGUS are complementary methods for diagnosing pSS, and the two tests can be used in daily clinical practice.

This study had some limitations. First, this was a medical chart review study with all of the inherent limitations of such retrospective studies. Patients who completed the anti-SSA/Ro, USFR, Schirmer tests, and SGUS were included, which could lead to information bias. However, this large retrospective cohort study confirmed the cut-off of USFR in various age groups, Second, OSS data were insufficient for our study. OSS was performed at only one institution, according to the judgment of an ophthalmologist. Third, using clinical diagnosis performed by experienced rheumatologists for pSS, instead of expert consensus, as the gold standard for evaluating the ACR/EULAR classification criteria. Further large-scale prospective studies including a wide range of patient characteristics are needed to confirm our findings.

In conclusion, the cut-off of USFR ≤ 0.1 mL/min was optimal, irrespective of age. Using SGUS can improve the capacity of ACR/EULAR criteria for classification as pSS by supplementing the USFR, especially in middle-aged patients.

## Data availability statement

The data underlying this article will be shared by the corresponding author upon reasonable request.

## Ethics statement

The studies involving human participants were reviewed and approved by Institutional Review Board for Human Research of Soonchunhyang University Seoul Hospital (2020-02-004) and Konkuk University Medical Center (1010776). Written informed consent for participation was not required for this study in accordance with the national legislation and the institutional requirements.

## Author contributions

K-AL: contributed to the conceptualization, methodology, and writing of the original draft preparation. S-HK: data curation and software. H-RK: data curation and validation. H-SK: supervision and writing, which consisted of reviewing and editing. All authors contributed to the article and approved the submitted version.

## Funding

This research was supported by Soonchunhyang University and by a grant of the Korea Health Technology R&D Project through the Korea Health Industry Development Institute (KHIDI), funded by the Ministry of Health and Welfare, Republic of Korea (Grand Number HI21C1888).

## Conflict of interest

The authors declare that the research was conducted in the absence of any commercial or financial relationships that could be construed as a potential conflict of interest.

## Publisher's note

All claims expressed in this article are solely those of the authors and do not necessarily represent those of their affiliated organizations, or those of the publisher, the editors and the reviewers. Any product that may be evaluated in this article, or claim that may be made by its manufacturer, is not guaranteed or endorsed by the publisher.
